# Changing Dietary Habits: The Impact of Urbanization and Rising Socio-Economic Status in Families from Burkina Faso in Sub-Saharan Africa

**DOI:** 10.3390/nu14091782

**Published:** 2022-04-24

**Authors:** Silene Casari, Monica Di Paola, Elena Banci, Salou Diallo, Luca Scarallo, Sara Renzo, Agnese Gori, Sonia Renzi, Monica Paci, Quirijn de Mast, Tal Pecht, Karim Derra, Berenger Kaboré, Halidou Tinto, Duccio Cavalieri, Paolo Lionetti

**Affiliations:** 1 Gastroenterology and Nutrition Unit, Meyer Children’s Hospital, 50139 Florence, Italy; silene.casari@gmail.com (S.C.); monica.dipaola@meyer.it (M.D.P.); luca.scarallo@gmail.com (L.S.); sara.renzo@meyer.it (S.R.); monica.paci@meyer.it (M.P.); 2 Dietetics Unit, Meyer Children’s Hospital, 50139 Florence, Italy; elena.banci@meyer.it; 3 Institut de Recherche en Sciences de la Santé-Clinical Research Unit of Nanoro (IRSS-URCN), Nanoro 18, Burkina Faso; saloudiallo89@yahoo.fr (S.D.); kderra@crun.bf (K.D.); kaboreberenger@gmail.com (B.K.); halidoutinto@gmail.com (H.T.); 4 Department of Neurology, Pharmacology, Psychology and Child Health (NEUROFARBA), University of Florence, 50139 Florence, Italy; agnese.gori@unifi.it (A.G.); sonia.renzi@unifi.it (S.R.); 5 Department of Internal Medicine, Radboud Center for Infectious Diseases, Radboud University, 6500 Nijmegen, The Netherlands; quirijn.demast@radboudumc.nl; 6 Genomics and Immunoregulation, Life and Medical Sciences (LIMES) Institute, University of Bonn, 53127 Bonn, Germany; talpecht@uni-bonn.de; 7 Department of Biology, University of Florence, Sesto Fiorentino, 50019 Florence, Italy; duccio.cavalieri@unifi.it

**Keywords:** urbanization, rural diet, Western diet, sub-Saharan Africa, Burkina Faso, fiber intake

## Abstract

(1) Background: Sub-Saharan Africa is experiencing the fastest urbanization worldwide. People in rural areas still have a traditional and rural lifestyle, whereas the Westernization of diet and lifestyle is already evident in urban areas. This study describes dietary habits of families in Burkina Faso living at different levels of urbanization. (2) Methods: Data on lifestyle, socio-economic conditions, health status and anthropometry were collected from 30 families living in rural villages, a small town and the capital city. A food frequency questionnaire and a 24 h recall diary were used to estimate dietary habits and macronutrients intake. (3) Results: The urban cohort showed a more diversified diet, with a higher intake of animal protein and, especially in children, a higher consumption of simple sugars. Fiber intake was significantly higher in the rural and semi-urbanized cohorts. As expected, overweight and obesity gradually increased with the level of urbanization. In semi-urbanized and urban families, we observed coexistence of under- and over-nutrition, whereas in rural families, a portion of children were wasted and stunted, and adults were underweight. (4) Conclusions: These three cohorts represent a model of the effect on diet of rural-to-urban migration. Rural diet and traditional habits are replaced by a Western-oriented diet when families move to urbanized areas. This dietary transition and increased socio-economic status in newly developing urban areas have a major impact on disease epidemiology, resembling the past evolution in Western countries.

## 1. Introduction

Industrialization in high-income countries has historically led to rural-to-urban migration, alongside a change in nutrition from mostly plant-based foods (vegetables, fruit, wholegrains, legumes) to hyper-caloric diets rich in total and saturated fats, cholesterol, animal protein, added salt and sugar and poor in fiber [1,2,3]. Currently, several regions in the world, including sub-Saharan Africa (sSA), are experiencing rapid urbanization, with a constant increase of people moving from rural to urban areas. This transition reflects socio-economic changes and a shift from subsistence farming to more specialized jobs. As a consequence, diet changes from a traditional towards a Western-like diet [4], with easier access to a greater food variety [5], including highly caloric and processed foods. Disease epidemiology is also radically changing. While the burden of infectious diseases remains high in many rural low-income areas, obesity and non-communicable diseases (NCDs) related to diet—once typical of the Western world—are rapidly increasing in the newly developing urban areas [6,7,8,9], accounting for almost half of all deaths and disability in low-to-middle-income countries [10]. In sSA, due to urban lifestyle and nutrition transition [11,12,13,14], many studies have described that NCDs, such as cardiovascular diseases, diet-related diseases, chronic kidney and respiratory diseases and cancers, will overtake infectious disease cases by 2030 [15,16,17,18]. These changes promote the adoption of behaviors that favor unhealthy diets and lifestyles, such as inadequate fruit and vegetable consumption and increased energy intake, harmful alcohol consumption, smoking and physical inactivity [19,20].

Burkina Faso, a country in West Africa, is in an early stage of demographic transition. The capital city of Ouagadougou and its surroundings represent the main urban area. Here, changes in lifestyle and dietary habits characteristic of urbanized populations have already taken place [21]. On the other hand, non-urban regions of Burkina Faso still present a traditional rural lifestyle mainly based on subsistence agriculture, with marginal contacts with the globalized world. A few studies have previously described dietary patterns of rural and urban children [22,23,24] and urban adults in Burkina Faso [25], but a detailed study of the current dietary habits of people living in environments at different levels of urbanization is missing.

Here, we describe the dietary and lifestyle habits of families of the same ethnicity living in areas at different phases of the rural-to-urban transition in Burkina Faso: rural villages, a semi-urbanized town (Nanoro) and the capital city Ouagadougou. The aim of the study was to investigate the effect of the rural-to-urban transition, focusing on households and family units as the center of a shared lifestyle and diet. 

## 2. Materials and Methods

### 2.1. Characteristic of the Region, Setting and Study Population

The current study was conducted in Burkina Faso from February 2019 to January 2020. The population of Burkina Faso is rapidly growing (20.9 million with a growth rate of 2.8% in 2019) [26] in particular with an annual urban growth rate remaining constantly high over recent years (4.9–6.7% per year in the decade 2009–2019) [27]. 

All individuals were enrolled during the dry season, which in Burkina Faso runs approximately from October to April, with variations depending on the region. In particular, the rural and the semi-urbanized cohorts were enrolled during February 2019, and the urban cohort in January 2020.

**Figure 1:**
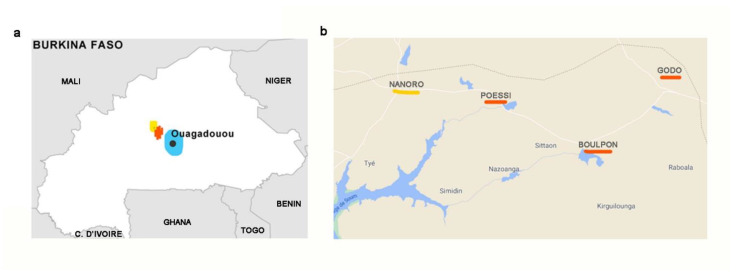
Map of Burkina Faso and areas of the three enrolled cohorts. (**a**) In blue: Urban area of the capital city Ouagadougou; in yellow: The small town of Nanoro (initial level of urbanization); in red: Rural area. (**b**) Detailed map. In red, the three rural villages of Boulpon, Godo and Poessi; in yellow, the town of Nanoro.

Thirty families were enrolled from three areas at different levels of urbanization: (i) Three rural villages from Boulkiemde province: Boulpon (geographic coordinates 12°39′00′ N 2°4′00″ W), Godo (12°01′00″ N−2°34′00″ W), and Poessi (12°20′14.55″ N 2°13′25.20″ W); (ii) the small town of Nanoro (Boulkiemde province, 12°41′ N 2°12′ W), with a population of 7542 inhabitants; (iii) the Capital city, Ouagadougou (12°21′26″ N 1°32′7″ W), with 2453 million inhabitants (Figure 1).

**Figure 2:**
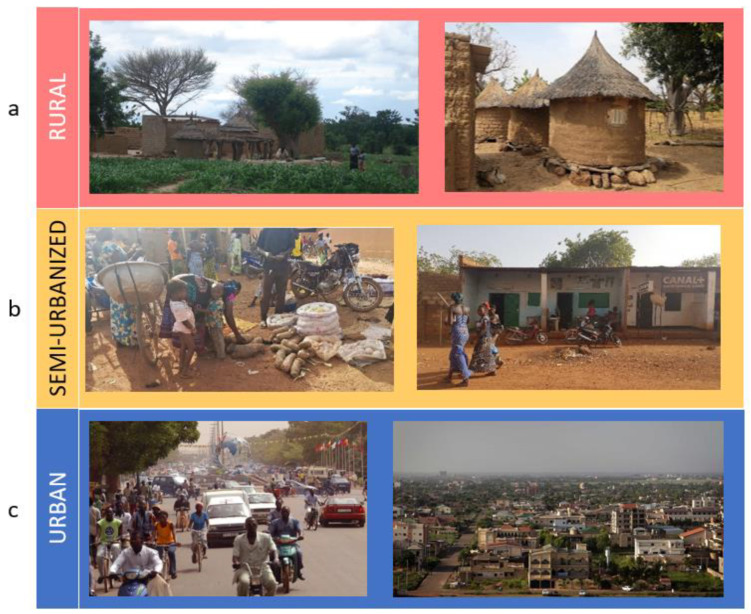
Life in Burkina Faso. (**a**) Rural village (Boulpon), (**b**) semi-urbanized area (Nanoro town), (**c**) the capital city Ouagadougou. Personal photographs taken by Paolo Lionetti and Silene Casari.

The Nanoro Health and Demographic Surveillance System (HDSS) [28], a surveillance organ established by the Clinical Research Unit of Nanoro (CRUN), randomly selected the households and families living in the three areas (rural, semi-urbanized and urban) based on different socio-economic conditions, lifestyle, diet, hygiene and sanitation. In rural Burkina Faso, most people live from subsistence agriculture. Rural villages are composed of clusters of huts built using soil, wood and straw (Figure 2). Rural families are on average poor; children are at high risk of infectious diseases and malnutrition, especially during the lean season between harvests (usually between May and September). Electricity is unavailable and the population gets water from wells or dams. Daily foods include cereals (millet, sorghum, maize), legumes (cowpeas, locally called Niébé, *Vigna unguiculata*) and vegetables (baobab leaves, wild herbs, tomatoes, peppers or Nerè, the fruit of *Parkia biglobosa* tree). Families usually share a single main meal made of tô, a thick porridge obtained from ground millet, sorghum or corn served with a sauce made from local vegetables, herbs, soumbalà (a condiment made of Néré seeds) and peanuts. Sometimes tô is supplemented with a small piece of meat (mainly goat, sheep and chicken, less frequently beef and pork) or dried fish. Young children are usually fed bouille, a liquid porridge made with millet, soy, peanuts and sugar.

In the town of Nanoro (Figure 2), there are urban groupings of small brick houses. In this semi-urbanized area, a few families have access to private sources of water, but all of them use public wells. Electricity is commonly absent in the houses, and all households host livestock in their courtyards. In this area, children are at risk of infectious disease and at low–medium risk of malnutrition. Diets are still predominantly plant-based but different products, such as cereal flour, rice, legumes, fruit, meat and dried fish can be easily found at the bi-weekly local market, together with foods from neighboring regions. People occasionally consume highly processed and Western-like food.

In the capital city of Ouagadougou (Figure 2), select wealthy families live in concrete or brick buildings with access to a private water source and electricity. With regard to diet, these families have a typical Burkinabe diet, rich in whole grain cereals, such as millet, sorghum and maize, legumes and local fruit and vegetables. The rapid urbanization of this area is bringing with it an increased wealth, with greater food availability and variety of processed food (such as fruit juices, snacks, sweets and bakery products) from supermarkets.

A total of 159 individuals (parents and children) were enrolled from three cohorts as follows:

- Rural cohort: 10 households (*n* = 54 individuals) living in the rural villages of Boulpon (*n* = 4 households), Godo (*n* = 3) and Poessi (*n* = 3).
- Semi-Urbanized cohort: 10 households (*n* = 55 individuals) from Nanoro.
- Urban cohort: 10 households (*n* = 50 individuals) living in the capital city of Ouagadougou selected among wealthy households.

Inclusion criteria for the families were as follows: (i) Households composed of a biological father and mother(s) living in the same household with at least one child, aged between 2 and 18 years, in apparently good health; (ii) belonging to the Mossi ethnic group, the main ethnic group in Burkina Faso. In order to exclude acute illnesses at the time of enrolment, individuals who had had fever (>38.5 °C) in the previous 72 h were excluded.

### 2.2. Ethics

Ethical clearance was obtained from the National Ethics Committee of Burkina Faso in August 2018 (reference number 2018-8-104). Enrolled adults gave informed consent. For children under 18, consent was given by primary caregivers. Confidentiality was maintained by allocating an identification code for each participant used on all questionnaires.

### 2.3. Data Collection and Nutritional Analyses

A questionnaire on lifestyle, health status and dietary habits was administered on site to each recruited individual. In cases of illiteracy, a member of local trained staff conducted a direct interview. The questionnaire was written in French and translated into Moorè (local language of Mossi ethnicity). Support of the local staff was ensured. Collected data were entered into a database. The dietary section of the questionnaire was tailored to the study population and included a semi-quantitative food frequency questionnaire (FFQ) and a 24-h diet recall, from which daily energy and macronutrient intakes were estimated. Food frequency referred to the week prior to the interview and regarded all the main local foods, as well as processed and refined foods. For every food item, pictures of the portion size of dishes (small–medium–large) were shown to estimate the amount of food consumed by each individual. In Supplementary Material Table S1, the estimated quantities for each portion size, divided for age groups, are reported. For analytical food composition, FAO/INFOODS Food Composition Databases were used [29].

### 2.4. Dietary Diversity Score

Data from the 24-h diet recall and from the questionnaire of weekly frequency consumption for each individual were used to calculate a dietary diversity score (DDS), a qualitative measure of the variability of food consumption, based on FAO guidelines for dietary diversity [30]. It is considered an indicator of macro- and micronutrient adequacy for all age groups [30,31,32,33,34]. Food items consumed during the seven days prior to the interview were categorized into 12 food groups (cereals, white tubers and roots, vegetables, fruits, meat, eggs, fish and seafood, legumes, nuts and seeds, milk and milk products, oils and fats, sweets, spices and condiments). A score equal to 1 was assigned for every food group included in the individual’s diet without considering a minimum intake for the food group. The sum of the scores represents each individual’s diet diversity score (minimum 0 and maximum 12).

### 2.5. Anthropometry and Classification of Nutritional Status

Trained fieldworkers collected anthropometry measurements. Individuals <18 years of age were considered children. Weight and height were measured for all enrolled individuals, with participants wearing light clothing and no shoes. Data on the nutritional status of children (<18 years) were derived from WHO databases [35]. These data include measurements of stunting (height-for-age, Z score < −2 SD), wasting (weight-for-height, Z score < −2 SD, if under 5 years; BMI < −2 SD, if 5–17 years, for moderate acute malnutrition, weight-for-height, Z score < −3 SD, if under 5 years; BMI < −3 SD, if 5–17 years for severe acute malnutrition) and overweight (weight-for-age Z-score > +2 SD, if under 5 years; BMI > +2 SD, if 5–17 years) [36]. For adult nutritional status, BMI was calculated and classified according to BMI cut points for underweight, overweight and obesity (<18.0 kg/m^2^, ≥25.0 kg/m^2^, and ≥30.0 kg/m^2^, respectively), based on the standard classifications of nutritional status as reported in the WHO website [37].

### 2.6. Statistical Analysis

Data were managed and analyzed using Microsoft Office Excel 2016 and IBM SPSS Statistic 20 software (IBM Corp., Armonk, NY, USA). Categorical variables were described as frequency and percentages. Continuous variables were evaluated for normal distribution using Kolmogorov–Smirnov goodness-of-fit test. Normally distributed continuous variables were presented as mean +/− standard deviations (SDs). Non-normally distributed variables were presented as medians (interquartile ranges (IQRs)). Univariate analyses were carried out on questionnaire results about lifestyle, health, anthropometric measures and dietary habits. Analyses of statistical differences among categorical variables were carried out using Chi-square or Fisher Exact test, where appropriate. We performed unpaired Student *t* test and Wilcoxon–Mann–Whitney tests to assess disparities among quantitative continuous variables. All statistical tests were two sided and *p* < 0.05 was considered as the statistically significant threshold.

## 3. Results

### 3.1. Characteristics of the Three Burkinabè Cohorts

In this study, 159 individuals (children and adults) took part belonging to households and families living in three areas at different level of urbanization (rural villages, semi-urbanized area around Nanoro town and the capital city Ouagadougou). The age range was from 0.7 to 77.1 years (mean age 24.7 years ± 20.4 standard deviation). Demography and socio-cultural status of the study populations are shown in Table 1. Living conditions of the households enrolled in the three different environments, including water source, cooking energy, light source, and coexistence with livestock are reported in Supplementary Material Table S2. Parents in all three cohorts were aged > 18 years. Enrolled families in the rural and semi-urbanized areas were large, composed of a man with one or more wives and their respective children, all sharing the same household. Polygamy is widely practiced (100% in rural and 60% in semi-urbanized households; Supplementary Material Table S2). In the capital city, the selected wealthy families were all monogamous (Supplementary Material Table S2).

Christian and Muslim religions are widespread in the three areas, especially in the urban cohort. However, in the rural villages, traditional animism is still the most practiced religion (Table 1). As for education, in the rural cohort, men and women are mostly illiterate, and children usually do not attend school, often because of the long walking distance from home. In the semi-urbanized cohort, education of adults is low, especially among women (77.8% and 81.8% of male and female caregivers, respectively, did not receive any education). All children of the semi-urbanized cohort attend school, also due to the proximity of school from their home. In the urban cohort, education and schooling rates are high (88.9% and 80% of male and female adults, respectively, completed at least a primary cycle education, and 85.7% of enrolled children attended school (Table 1)).

With regard to occupations, agriculture is the main activity in the rural and semi-urbanized cohorts, especially for women (100% of women are housewives and farmers in both cohorts), while different manual jobs can be found among men. In the capital city of Ouagadougou, adults work mostly in professional or specialized jobs (44.4% of males and 40% of females).

Concerning habitual alcoholic beverage consumption, fermented beverages are very popular in Burkina Faso. “Dolo”, a low alcoholic grade beverage (about 4–6% *v*/*v*) produced by fermentation of sorghum, is commonly consumed in rural areas [38]. Adults of the rural cohort reported habitual alcohol consumption (62%, 18/29), mostly based on dolo (55.2%, 16/29; Table 1). In the semi-urbanized and urban cohorts, dolo consumption was lower (17.4%, 4/23, for semi-urbanized and 12.5%, 3/24, for urban adults). In the capital city adults consume mainly commercial alcoholic drinks, such as beer, wine or spirits (72.7%, 8/24). Regarding smoking, a small proportion of adults of the semi-urbanized and urban cohorts declared they usually smoke cigarettes (Table 1).

In urban areas, transport and mobility are mainly via motorized means (66.7% of the adult individuals declared they use scooters and cars; Table 1) and lifestyle is predominantly sedentary. In rural and semi-urbanized areas, people move mainly on foot or by bike (89.7% and 69.6% of the adults, respectively; Table 1), they have an active lifestyle, also due to heavy physical work.

### 3.2. Energy and Macronutrient Intake Estimation Per Area

**Figure 3:**
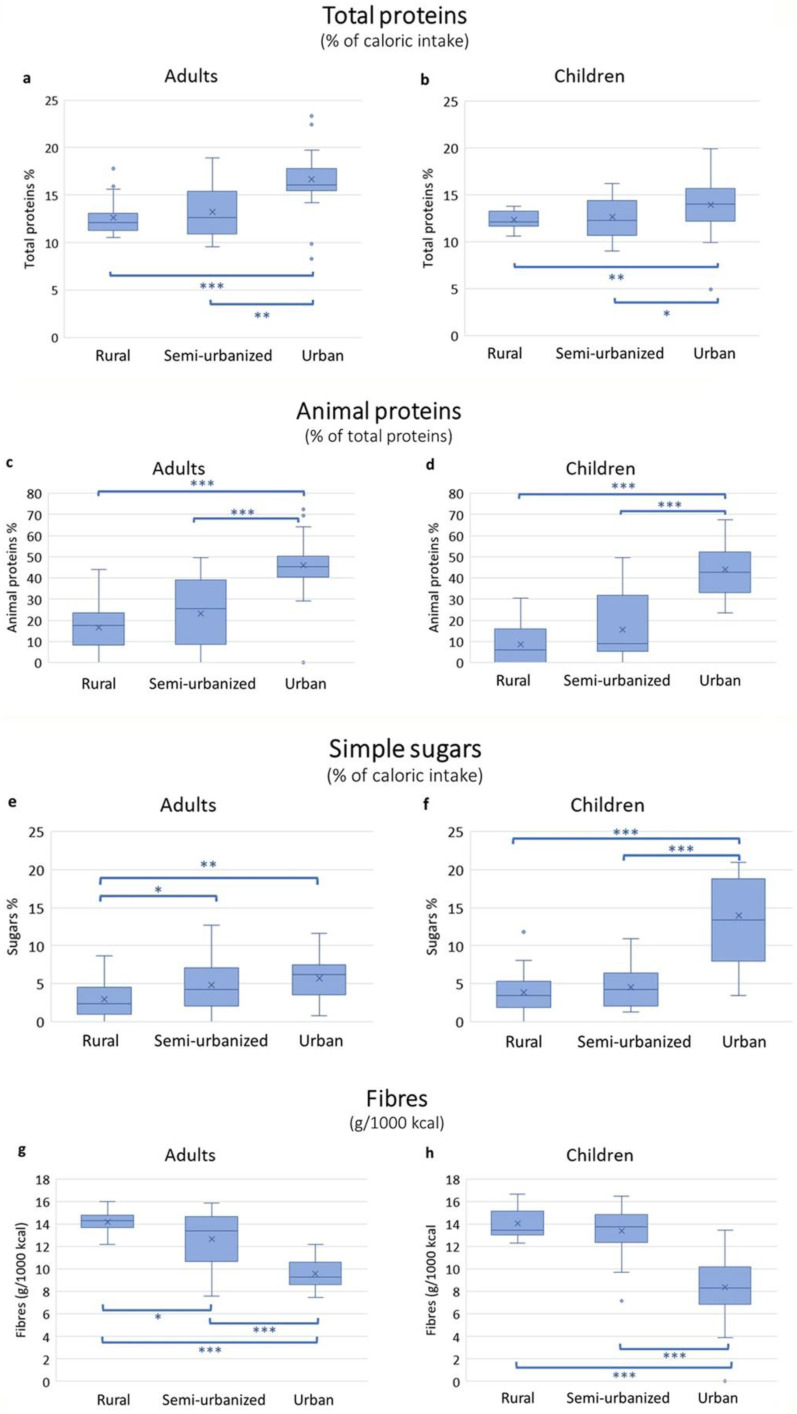
Macronutrient intake in adults and children in the rural, semi-urban and urban areas. The box plots represent median with interquartile range. Total proteins expressed as percentage of total caloric intake in adults (**a**) and children (**b**). Animal proteins as percentage of total proteins in adults (**c**) and children (**d**). Simple sugars expressed as percentage of total caloric intake in adults (**e**) and children (**f**). Fiber intake expressed as grams per 1000 kcal in adults (**g**) and children (**h**). Statistically significant by Mann–Whitney test *** *p* ≤ 0.001, ** *p* ≤ 0.01, * *p* ≤ 0.05.

Energy and macronutrient intake for adults and children are reported in Supplementary Material Tables S3 and S4, respectively. Calories, carbohydrates and fat intake did not differ significantly among the families in the three areas. In contrast, the intake of protein, fibers and simple sugars differed significantly (Figure 3). Total protein intake was significantly higher among the urban participants compared to the other two groups. Almost half of the total protein amount was of animal origin in the urban cohort, both in adults and children. Among adults, the consumption of simple sugars did not differ significantly between urban and semi-urbanized participants, but it was significantly lower in rural participants. Among children, those in the urban setting had a significantly higher intake of simple sugars compared to children from other areas. Fiber intake in adults was highest in those from the rural area, followed by those in the semi-urbanized area and the capital. Sixty-nine percent of rural adults reached the recommended fiber intake (12.6–16.7 g/1000 kcal for adults according to EFSA [39]), 26% in the semi-urbanized group and 0% in the urban adults. In children, fiber intake was similarly high in both rural and semi-urbanized cohorts (100% and 97% of children reached the recommended intake of 8.4 g/1000 kcal [39]), but significantly lower in the urban children (50% of children were not reaching the recommended fiber intake; Supplementary Material Figure S1).

### 3.3. Food Frequency Consumption and Estimation of Dietary Diversity

**Figure 4:**
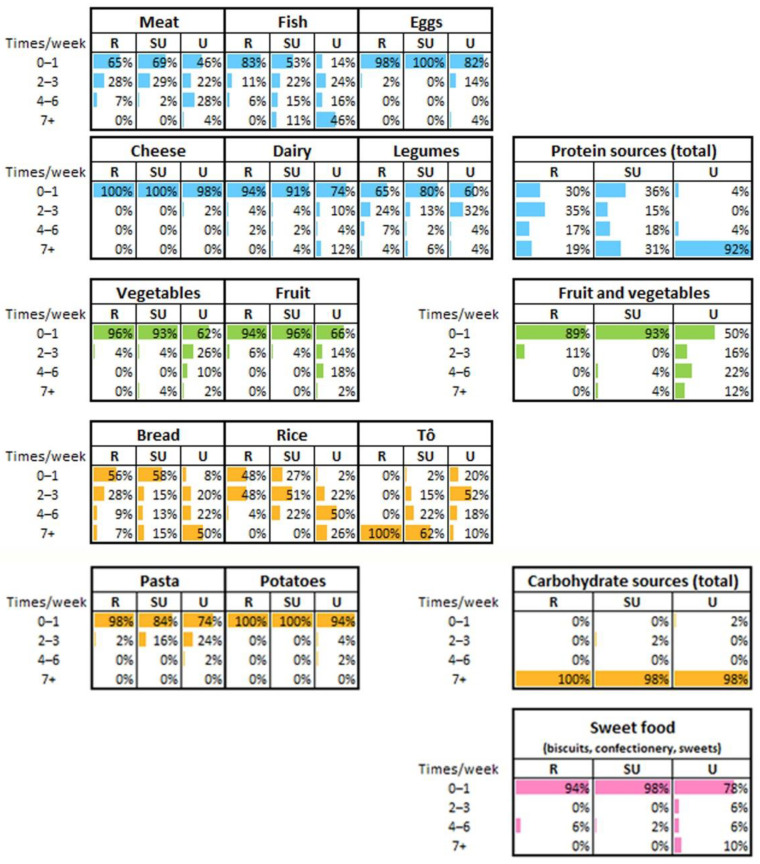
Weekly frequency of food consumption. The frequency of consumption of the categorized food items was referred to the seven days prior to the interview. Data are referred to all the enrolled individuals, without distinction of age. R = rural; SU = semi-urbanized; U = urban cohorts. Percentages may not add up to exactly 100% due to rounding. Right panel: Table of the total protein sources showing the proportion of individuals from the three cohorts that consume food rich in proteins (such as meat, fish, egg, cheese, dairy and legumes). Similarly, table of fruit and vegetables showing the proportion of individuals consuming weekly both fruits and vegetables; table of total carbohydrate sources showing the proportion of individuals consuming weekly foods rich in carbohydrates (such as bread, rice, to, pasta and potatoes); table of sweet food showing the proportion of individuals consuming weekly foods rich in simple sugars.

Weekly frequency of consumption of major food items (such as meat, fish, eggs, cheese, dairy, main carbohydrate sources, fruit and vegetables, sweet foods) is reported in Figure 4. Food items that contribute to protein intake are consumed daily by 19% of rural participants, 31% of semi-urbanized participants and 92% of urban participants. In rural families, protein sources are mainly legumes and, sometimes, small quantities of meat or fish, whereas eggs or dairy are not consumed. In contrast, urban participants showed a more diversified diet with more frequent consumption of fish, meat, eggs and dairy during the week compared to the rural cohort. In addition, fruits and vegetables are consumed more often among the urban participants than the rural ones. Among carbohydrate sources, to is the main rural meal, while bread, rice and pasta are frequent alternatives in the urban cohort. The semi-urbanized participants showed intermediate frequency of consumption for almost all food groups. Interestingly, 10% of urban participants reported daily consumption of sweet items (including industrial cookies, confectioneries and sweets), while the rural and semi-urbanized cohorts rarely consume them. None of the rural participants consumed meals at restaurants and only 2% of semi-urbanized participants had consumed more than two restaurant meals in the week preceding the interview. In contrast, 22% of urban participants consumed restaurant meals ranging from twice to more than seven times per week. A small proportion of rural individuals (8%) declared having consumed meals at a local market (Supplementary Material Table S5). Food items purchased from supermarkets were consumed only by urban participants.

**Figure 5:**
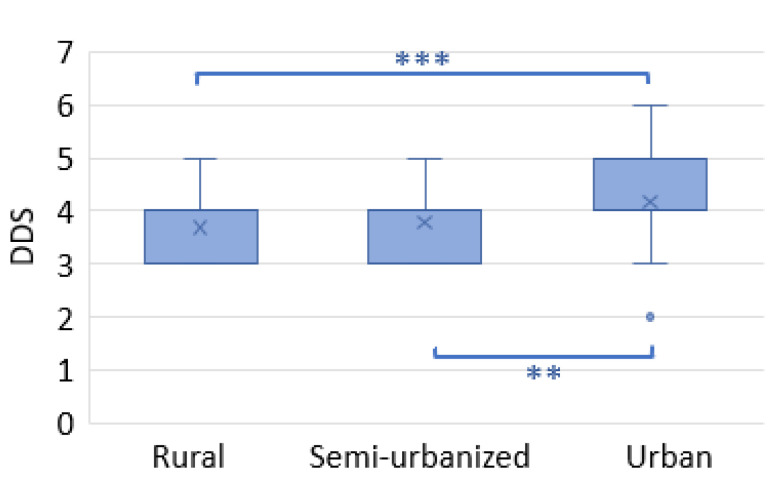
Dietary diversity score (DDS). The box plots show the median and interquartile range (IQR). “x” is the mean and blue dots are outliers. *** *p* < 0.001 ** *p* < 0.01 (Mann–Whitney test).

In order to assess differences in food variety consumed among the three cohorts, the dietary diversity score (DDS) was calculated. The DDS was similar across the rural and semi-urban families, but significantly higher in urban families (Figure 5).

### 3.4. Nutritional Status Changes across the Cohorts

**Figure 6:**
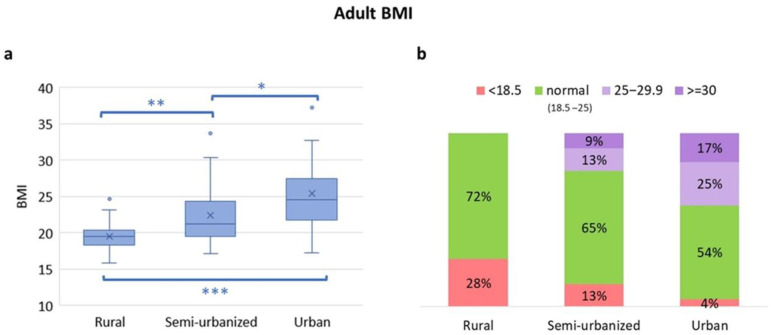
Comparison of the BMI categories among adults from rural, semi-urban and urban areas. (**a**) BMI distribution. Boxplots show median and interquartile range. “x” is the mean and blue dots are outliers. Statistically significant by Mann–Whitney test *** *p* ≤ 0.001, ** *p* ≤ 0.01, * *p* ≤ 0.05. (**b**) Proportion of adults with underweight (BMI < 18.5 kg/m^2^), normal weight (BMI 18.5–25 kg/m^2^), overweight (BMI 25–29.9 kg/m^2^) and obesity (>30 kg/m^2^) across the areas.

**Figure 7:**
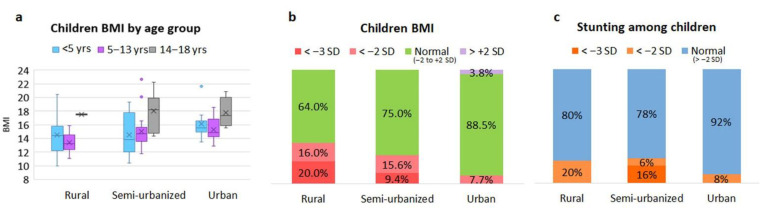
Comparison of BMI categories among children from the rural, semi-urban and urban areas. (**a**) BMI by age group, boxplots show median and interquartile range (IQR). “x” is the mean and blue dots are outliers (**b**) BMI distribution. BMI < −2 SD defines acute malnutrition; <−3 SD severe acute malnutrition. For children < 5 years, SD of weight-for-height was considered instead of BMI. (**c**) Prevalence of stunting among children; <−2 SD as moderate stunting, <−3 SD as severe stunting. yrs, years old.

The nutritional statuses of adults and children from the different areas are shown in Figure 6 and Figure 7, respectively. The BMI in adults showed a gradient with rural adults having the lowest BMI and urban adults the highest (Figure 6a,b).

In Supplementary Material Table S6, adults’ BMI score divided by gender is reported. As expected, overweight and obesity are absent in the rural cohort, whereas an increasing proportion of adults from the semi-urbanized or urban area are overweight (13% and 25%, respectively) or obese (9% and 17%, respectively). In contrast, in the rural cohort, 28% of adults are underweight. Among children, standard deviations of BMI-for-age (5–18 years) and weight-for-height (under 5 years) were calculated to obtain the proportion of children who were underweight or suffered acute malnutrition (Figure 7a,b). Thirty-six percent of rural children were malnourished (16% with moderate acute malnutrition, MAM, and 20% with severe acute malnutrition, SAM). This proportion was lower in the semi-urbanized (15.6% MAM, 9.4% SAM) and in the urban (7.7% MAM, 0% SAM; Figure 7a) children. With regard to chronic malnutrition, there were no differences in the prevalence of stunting (defined as a height-for-age below −2 SD) between the rural and semi-urbanized children (20% and 22%, respectively). In contrast, the proportion of stunted children was reduced (8%) in urban children (Figure 7c).

**Figure 8:**
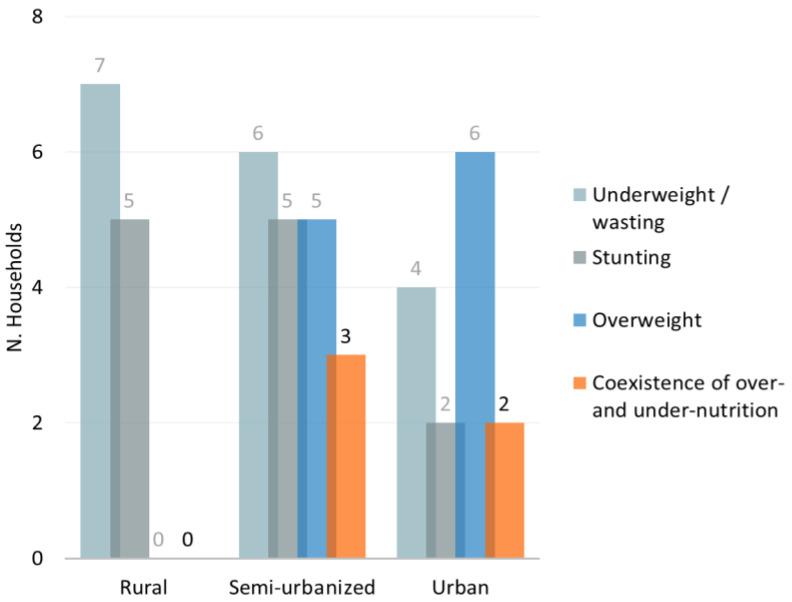
Double burden of malnutrition. Distribution of different forms of malnutrition (under- or overweight) within families. The number of households with at least one family member affected by underweight, stunting or overweight was reported.

Finally, in the semi-urbanized and urban families we observed a co-presence of family members affected by either under- or over-nutrition (three out of 10 households and in two out of 10, respectively; Figure 8). On the other hand, in the rural and semi-urbanized households, no adult or child was overweight, but, generally, at least one family member (child or adult) was underweight/wasted or stunted, when compared to urban families (Figure 8).

## 4. Discussion

This study describes dietary habits and lifestyle of families belonging to the same ethnic group and living at different stages of urbanization in Burkina Faso. In accordance with previous reports [40,41,42,43], we observed pronounced socio-cultural and economic changes, associated to different dietary patterns, across families living in rural areas, in semi-urban areas and in the capital city. The main changes observed were in (i) family composition: Polygamy in the rural and semi-urbanized cohorts versus monogamy in the urban area; (ii) education: High illiteracy in rural areas, especially among women; (iii) employment: Manual and field workers in the rural and semi-urbanized areas, more specialized jobs in the city; (iv) religion: Traditional animism in rural villages versus prevalence of monotheistic religion in the urban area; (v) dwelling typology: Traditional huts built with bundles of straw stored against their circular walls made of mud bricks without electricity and water versus brick houses with electricity and water supplies in the cities. 

The families in the selected areas represent a model that allows us to observe the effect of rapid urbanization and rural-to-urban transition, as well as the consequent socio-economic changes in dietary habits, lifestyle and health. It is worth noting that we selected households in the capital city with a medium–high income to observe the effect of this transition, similarly to what has happened in the past in the Western countries [27,44]. Therefore, dietary habits of low-income urbanized individuals were not investigated. 

As observed in our previous studies in children from Burkina Faso [22,23] and as described in other reports [1,2,3,4,11,14,19,20,21,45], the fast urbanization is bringing along a transition towards Western diets and lifestyles, increasing the distance from traditional rural areas where access to food is limited to local products and follows seasonal availability. In urban settings, supermarkets and restaurants provide access to a great variety of food items. Ultra-processed food, soft drinks and foods rich in additives, salt, sugar and saturated fats are easily accessible, determining a nutrition transition towards a Western-style diet. 

In our study, to collect information on dietary habits of the three cohorts, trained field staff administered both 24 h recall diary and a seven-day food frequency questionnaire (FFQ). The 24-h dietary recall and the FFQ are useful tools for dietary assessment and epidemiological purposes, and FFQ is often used in epidemiological studies to estimate long-term dietary exposure. These methods are mainly used for their applicability in large samples and their capability to categorize foods based on their intake [46,47]. Dietary assessment methods have potential limitations and they are hugely argued over in literature in order to keep attention on them and to ensure an adequate evaluation of dietary pattern and macronutrients intake in population surveys. A potential limitation of such dietary assessment methods is due to the risk of bias and under- or overestimation (mainly due to memory and portion size estimation) or caused by missing information when assessing habitual diets. A recent study in adolescents from Burkina Faso [48] validated the 24-h dietary recall with the support of the observed weighed food records for estimating nutrient intakes. In our study, a potential limitation of the dietary assessment methods could be the discrepancies in data collection between the 24-h dietary recall and the FFQ. Similarly, to support the compilation of both the diary and the questionnaire, pictures of size portions were shown to participants. Moreover, to limit as much as possible biases on the collected dietary information and to make sure that collected data were reliable, results of both 24-h dietary recall and of FFQ were compared in order to more precisely assess macronutrient intakes. 

In general, in the three cohorts of this study we observed that the intake of proteins, especially animal proteins, and simple sugars increases with the level of urbanization, while fiber intake showed an opposite trend. Similarly, others countries, such as Ghana in West Africa [49], or Tanzania in East Africa [45], or other world regions, such as rural China [50], have experienced fast urbanization and changes of dietary patterns across the rural-to urban transition. These studies described mainly a reduction of carbohydrates and fiber consumption and increased intake of animal protein and fat in urban populations.

Despite a low availability of fruits and vegetables outside the urban settings during the dry season (when the interview was conducted), Burkinabè people acquired dietary fiber from tô and/or bouille, both made with whole ground grains, and from plant-based ingredients used in the added sauce. Rural and semi-urbanized individuals showed higher fiber consumption compared to urbanites. These observations confirm in whole families what we previously reported in small cohorts of Burkinabè children living at different levels of urbanization compared to European children [22,23]. We previously showed that a fiber-rich diet, typical of the rural population, enriches the gut microbiota of bacterial species that are able to ferment dietary indigestible polysaccharides, producing short-chain fatty acids (SCFAs). These metabolites are well known for their anti-inflammatory role and for their contribution to the maintenance of health [51]. In contrast, a Western-style diet of urban people, rich in protein, simple sugars and fat and poor in fiber, has the potential to deeply influence gut microbiota composition, as well as the host’s health and development [52,53,54,55]. In recent years, a general increase in non-communicable conditions typical of Western industrialized countries, such as inflammatory bowel diseases (IBDs), type 2 diabetes, obesity, allergies and colorectal cancer [56] has been observed also in low-income countries, in association with improvement of socio-economic conditions and Western-like dietary habits. In the present study, the nutritional and anthropometric status changed significantly across the rural, semi-urban and urban areas. Overweight and obesity in children and young adults are rapidly increasing all over the world, albeit at different rates, depending on geographic location and following different development models [57]. Similarly, rural areas in China have experienced the co-existence of overweight and obesity, undernutrition and micronutrient deficiencies associated to changes of dietary habits [50,58]. In the past, childhood overweight was considered an exclusive problem in high-income countries but we are currently witnessing an increase even in low- and middle-income countries (LMICs), and especially in urban areas [59]. The World Health Organization (WHO) estimated that 39 million children under the age of 5 and 340 million children and adolescents between 5 and 19 years are overweight or obese [56]. Overweight and obesity in these age groups deserve particular attention, as they represent a particular risk factor linked to the onset of numerous chronic diseases, such as diabetes, cardiovascular diseases and some types of cancer [60]. Furthermore, pre-adolescent and adolescent obesity is considered a strong predictor of obesity in adulthood [61]. It has been calculated that more than a third of children and about half of adolescents who are overweight maintain this condition when they become adults [62], with all the consequences that this entails in terms of health and social costs. On the other hand, malnutrition is also still a major public health problem in LMICs. Across the areas in our study, the prevalence of underweight and acute malnutrition (wasting) decreased with the increase in urbanization level. The prevalence of child stunting was similar in the non-urban children, but much lower in the capital city. In addition to diet, the high prevalence of recurrent infections, including malaria, in the rural environments compared to urban areas is an important factor [63]. Infections can cause rapid weight loss in children and, in the long term, threaten the achievement of full development. Prolonged undernutrition leads to stunting, a severe condition with long-term consequences such as impaired neurocognitive development. The last nutritional survey in communities and host sites conducted in Burkina Faso in 2019 [64] reported a prevalence of severe wasting and wasting among children under 5 years of age of 1.0% and 8.1%, respectively, 23.8% of stunting and 1.6% of overweight. In our randomly selected families, even though children under 5 years represent a small proportion among the overall child group, we observed that the prevalence of severe wasting, moderate wasting and stunting were significantly higher in the rural children compared to urban children. In contrast, in the urban cohort, wasting and stunting were not detected in children under 5 years of age, whereas one child out of ten was obese.

The “double burden of malnutrition” refers to “the coexistence of undernutrition along with overweight, obesity or diet-related NCDs, within individuals, households and populations, and across the life-course” [65]. This condition is often observed as a side-effect of urbanization, demographic transition, changes in occupational structure and shifts in patterns of diet and physical activity [65,66,67]. As previously described in literature [68], in some urban families enrolled in this study, we found the coexistence of under- and over-nutrition. This condition was not observed in rural families where we reported various forms of undernutrition. However, it is interesting to highlight how the nutrition transition starts well before full-fledged urbanization. In fact, families from the semi-urbanized cohort showed a higher presence of the double burden within the same household, emphasizing the risk of areas at an early stage of urbanization paying the double price of the consequences of under- and over-nutrition. Nonetheless, nutritional interventions in these areas are still focused almost uniquely on undernutrition.

## 5. Conclusions

Burkina Faso is a low-income sub-Sahelian country at an early stage of urbanization. Rapidly urbanizing areas, like the capital city of Ouagadougou, are quickly showing the effects of the globalization of food systems and the westernization of both diet and lifestyle. This phenomenon is consequently associated with the increase in conditions typical of the globalized world, including overweight, obesity and other NCDs. We are aware that the health outcomes of urbanized populations and tailored interventions to break the emergent epidemic may not be fully identified in a short period of time. In the future, the complex interactions among urbanization, environment, diet, socio-economic and cultural status requires longitudinal population studies to deeply describe dietary transition and its evolution over time, as well as health determinants and outcomes in African urban areas. On the other hand, rural areas of sub-Saharan Africa continue to lag behind, especially in terms of education and health, with high levels of illiteracy and high prevalence of infections and different forms of under-nutrition like child wasting and stunting or adult underweight.

## Figures and Tables

**Table 1:** Demographic and socio-cultural characteristics of study participants.

	Rural (R) *n*. (%)	Semi-Urbanized (SU) *n*. (%)	Urban (U) *n*. (%)
Participants (*n* = 159)	54 (34.0)	55 (34.6)	50 (31.4)
Fathers/mothers (*n* = 26/40)	8 (30.8)/19 (47.5)	9 (34.6)/11 (27.5)	9 (34.6)/10 (25.0)
Offspring (*n* = 93)	27 (29.0)	35 (37.6)	31 (33.4)
Children Males/females (*n* = 44/49)	14 (31.8)/13 (26.5)	16 (36.4)/19 (38.8)	14 (31.8)/17 (34.7)
**Age distribution (years)**			
<5 (*n* = 27)	11 (40.7)	6 (22.3)	10 (37.0)
5–13 (*n* = 43)	12 (27.9)	19 (44.2)	12 (27.9)
14–18 (*n* = 13)	2 (15.4)	7 (53.8)	4 (30.8)
>18–77 (*n* = 76)	29 (38.1)	23 (30.3)	24 (31.6)
Parents/caregivers (*n* = 66)	27 (40.9)	20 (30.3)	19 (28.8)
**Religion of Parents/caregiver**			
Muslim	6 (22.2)	7 (35)	8 (42.1)
Christian Catholic	7 (25.9)	5 (25)	9 (47.4)
Christian Protestant	2 (7.5)	6 (30)	2 (10.5)
Traditional (animist)	12 (44.4)	2 (10)	0 (0)
**Education level of parents/caregivers**			
Males			
No school education	8 (100)	7 (77.8)	1 (11.1)
Primary school	0 (0)	0 (0)	3 (33.3)
Intermediate	0 (0)	1 (11.1)	3 (33.3)
High or more	0 (0)	1 (11.1)	2 (22.2)
Females			
No school education	18 (94.7)	9 (81.8)	0 (0)
Primary school	1 (5.3)	2 (18.2)	2 (20)
Intermediate	0 (0)	0 (0)	6 (60)
High or more	0 (0)	0 (0)	2 (20)
**Occupation of parents/caregivers**			
Males			
Manual worker	8 (100)	8 (88.9)	4 (44.4)
Professional	0 (0)	1 (11.1)	4 (44.4)
Retired	0 (0)	0 (0)	1 (11.2)
Females			
Housewife/farmer	19 (100)	11 (100)	4 (40)
Student	0 (0)	0 (0)	1 (10)
Manual worker	0 (0)	0 (0)	1 (10)
Professional	0 (0)	0 (0)	4 (40)
**School attendance among children at school age (6–16 y)**			
Attending	0 (0)	21 (100)	12 (85.7)
**Habitual alcohol consumption (parents/caregivers)**			
Alcoholic fermented products (e.g., dolo)	16 (55.2)	4 (17.4)	3 (12.5)
Alcoholic beverages (beer, wine, spirits)	2 (6.9)	0 (0)	8 (33.3)
**Smoking (parents/caregivers)**			
Smoking habit	0 (0)	1 (4.3)	1 (4.2)
**Means of transport of adults (>18 y)**			
Walk and/or bike	26 (89.7)	16 (69.6)	8 (33.3)
Scooter and/or car	3 (10.3)	7 (30.4)	16 (66.7)

## Supplementary Materials

The following are available online at https://www.mdpi.com/article/10.3390/nu14091782/s1, Figure S1: Proportion of individuals in the three cohorts, adults (upper panel) and children (bottom panel), who consume an adequate quantity of fibre based on the recommended standards. Table S1: Food portion sizes estimated for the main food items. An average standard composition of tô sauce was included in our calculation. For bouillie, an average composition was calculated (for 100 g of dry product: millet 60 g, soy 20 g, peanuts 10 g, sugar 9 g). Table S2: Living conditions of the households enrolled in the three different environments. Table S3: Estimation of macronutrients intake in adults of the three cohorts. Data are reported as median and interquartile range (IQR). Table S4: Estimation of macronutrients intake in children of the three cohorts. Data are reported as median and Interquartile range (IQR). For fibre, absolute amount in grams is not reported due to differences in age and weight of children. Table S5: Comparison of the weekly frequency of meals not prepared at home among the three cohorts, in particular food consumed at restaurant and food from a local market. Table S6: Adult BMI divided by gender. Median and IQR are reported.
