# Supplementary material for: Changing Dietary Habits: The Impact of Urbanization and Rising Socio-Economic Status in Families from Burkina Faso in Sub-Saharan Africa
Source: Nutrients. 2022 Apr 24;14(9):1782. doi: 10.3390/nu14091782 (PMC9104313; doi:10.3390/nu14091782)
Supplement: Supplementary file 1 [file nutrients-14-01782-s001.zip › nutrients-1664893-supplementary.pdf]

Supplementary Materials

Article

Changing Dietary Habits: The Impact of Urbanization and Rising Socio-Economic Status in Families from Burkina Faso in Sub-Saharan Africa

Silene Casari [1], Monica Di Paola [1], Elena Banci [2], Salou Diallo [3], Luca Scarallo [1], Sara Renzo [1], Agnese Gori [4], Sonia Renzi [4], Monica Paci [1], Quirijn de Mast [5], Tal Pecht [6], Karim Derra [3], Berenger Kaboré [3], Halidou Tinto [3], Duccio Cavalieri [7] and Paolo Lionetti [1,4] *

1 Gastroenterology and Nutrition Unit, Meyer Children's Hospital, 50139 Florence, Italy; silene.casari@gmail.com (S.C.); monica.dipaola@meyer.it (M.D.P.); luca.scarallo@gmail.com (L.S.); sara.renzo@meyer.it (S.R.); monica.paci@meyer.it (M.P.)

2 Dietetics Unit, Meyer Children's Hospital, 50139 Florence, Italy; elena.banci@meyer.it

3 Clinical Research Unit of Nanoro, (IRSS—URCN), B.P. 218 Ouagadougou 11, Burkina Faso; saloudiallo89@yahoo.fr (S.D.); kderra@crun.bf (K.D.); kaboreberenger@gmail.com (B.K.); halidoutinto@gmail.com (H.T.)

4 Department of Neurology, Pharmacology, Psychology and Child Health (NEUROFARBA), University of Florence, 50139 Florence, Italy; agnese.gori@unifi.it (A.G.); sonia.renzi@unifi.it (S.R.)

5 Department of Internal Medicine, Radboud Center for Infectious Diseases, Radboud University, 6500 Nijmegen, The Netherlands; quirijn.demast@radboudumc.nl

6 Genomics and Immunoregulation, Life and Medical Sciences (LIMES) Institute, University of Bonn, 53127 Bonn, Germany; talpecht@uni-bonn.de

7 Department of Biology, University of Florence, Sesto Fiorentino, 50019 Florence, Italy; duccio.cavalieri@unifi.it

* Correspondence: paolo.lionetti@unifi.it; Tel.: +39-055-5662950

This file includes:

Figure S1: Proportion of individuals in the three cohorts, adults (upper panel) and children (bottom panel), who consume an adequate quantity of fiber based on the recommended standards.

Table S1: Food portion sizes estimated for the main food items. An average standard composition of tô sauce was included in our calculation. For bouillie, an average composition was calculated (for 100 g of dry product: millet 60g, soy 20g, peanuts 10g, sugar 9g).

Table S2: Living conditions of the households enrolled in the three different environments.

Table S3: Estimation of macronutrients intake in adults of the three cohorts. Data are reported as median and interquartile range (IQR).

Table S4: Estimation of macronutrients intake in children of the three cohorts. Data are reported as median and Interquartile range (IQR). For fiber, absolute amount in grams is not reported due to differences in age and weight of children.

Table S5: Comparison of the weekly frequency of meals not prepared at home among the three cohorts, in particular food consumed at restaurant and food from a local market.

Table S6: Adult BMI divided by gender. Median and IQR are reported.

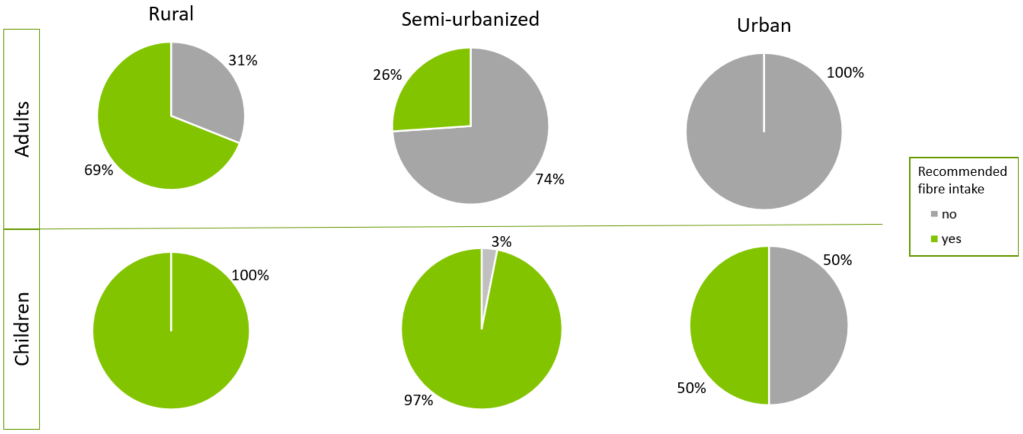

Supplementary Figure S1. Proportion of individuals in the three cohorts, adults (upper panel) and children (bottom panel), who consume an adequate quantity of fiber based on the recommended standards.

Supplementary Tables

Supplementary Table S1. Food portion sizes estimated for the main food items. An average standard composition of tô sauce was included in our calculation. For bouillie, an average composition was calculated (for 100 g of dry product: millet 60g, soy 20g, peanuts 10g, sugar 9g).

**Food portion size**	Children **2-6 y**	Children **7-14 y**	Adults **14+ y**
Tô *- Small	20 g	60g	100 g
Tô - Medium	40 g	80 g	130 g
Tô - Large	50 g	100 g	150 g
Tô sauce - Small	10g	30g	50 g
Tô sauce - Medium	20 g	40 g	60 g
Tô sauce - Large	30 g	50g	70 g
Meat	60 g	100 g	150 g
Fish	60 g	100 g	150 g
Eggs	60 g	100 g	100 g
Legumes (dried)	30g	40 g	60 g
Cheese (fresh)	60 g	100 g	100 g
Milk	200 ml	200 ml	200 ml
Yogurt	125 g	125 g	125 g
Oil	20 g	30 g	40 g
Soft drinks	330 ml	330 ml	330 ml
Sugar cube	5 g	5 g	5 g

* Tô quantities also apply to other carbohydrate sources (rice, bouille, etc); y=years old

Supplementary Table S2. : Estimation of macronutrients intake in adults of the three cohorts. Data are reported as median and interquartile range (IQR).

	**Rural** n. households (%)	**Semi-urbanized** n. households (%)	**Urban** n. households (%)
**Family composition**			
Polygamous families	10/10 (100)	6/10 (60)	0/10 (0)
**Livestock** (hens, pigs, goats, donkeys)	10/10 (100)	10/10 (100)	0/10 (0)
**Water source**			
Wells	10/10 (100)	10/10 (100)	0/10 (0)
Private tap	0/10 (0)	2/10 (20)	10/10 (100)
**Cooking energy**			
Coal, wood, millet stalks	10/10 (100)	10/10 (100)	3/10 (30)
Gas	0/10 (0)	0/10 (0)	8/10 (80)
**Light source**			
Wood and straw	4/10 (40)	0/10 (0)	0/10 (0)
Battery flashlight	9/10 (90)	7/10 (70)	0/10 (0)
Electricity	0/10 (0)	2/10 (20)	10/10 (100)

Some families have access to more than one water/energy/light source

Supplementary Table S3. Estimation of macronutrients intake in adults of the three cohorts. Data are reported as median and interquartile range (IQR).

**ADULTS**	Rural	Semi-urbanized	Urban
**Kcal tot**	1986 (448)	1711 (556)	1703 (460)
**Proteins** (% of caloric intake)	12.1 (1.8)	12.6 (4.8)	16.1 (2.3)
**Proteins** (g/kg)	1.11 (0.33)	1.00 (0.5)	1.15 (0.6)
**Animal proteins** (% of total proteins)	17.70 (13.4)	25.60 (30.3)	45.45 (9.1)
**Vegetable proteins** (% of total proteins)	82.30 (13.4)	74.40 (30.3)	54.55 (9.1)
**Fats** (% of caloric intake)	27.6 (6.1)	30.4 (4.4)	30.0 (6.3)
**Carbohydrates** (% of caloric intake)	58.4 (5.1)	57.2 (4.8)	53.7 (7.5)
**Sugars** (% of caloric intake)	2.4 (3.3)	4.2 (5.0)	6.2 (3.9)
**Fibers** (g)	28.2 (6.7)	21.7 (5.8)	16.3 (6.4)
**Fibers** (g/1000kcal)	14.3 (0.9)	13.4 (4.0)	9.3 (1.8)

Supplementary Table S4. Estimation of macronutrients intake in children of the three cohorts. Data are reported as median and interquartile range (IQR). For fiber, absolute amount in grams is not reported due to age and weight differences of children.

CHILDREN	Rural	Semi-urbanized	Urban
**Kcal tot**	1208 (519)	1245 (482)	1320 (564)
**Proteins** (% of caloric intake)	12.1 (1.4)	12.3 (3.6)	14.0 (3.3)
**Proteins** (g/kg)	0.4 (0.1)	0.3 (0.2)	0.4 (0.1)
**Animal proteins** (% of total proteins)	6.1 (15.5)	9.1 (26.4)	42.7 (17.4)
**Vegetable proteins** (% of total proteins)	93.9 (15.5)	90.9 (26.3)	57.3 (17.4)
**Fats** (% of caloric intake)	30.6 (4.3)	30.6 (4.7)	34.3 (6.8)
**Carbohydrates** (% of caloric intake)	57.4 (4.5)	56.3 (4.3)	51.6 (9.7)
**Sugars** (% of caloric intake)	3.4 (3.3)	4.2 (4.3)	13.4 (10.3)
**Fibers** (g/1000kcal)	13.5 (1.7)	13.8 (2.3)	8.4 (3.3)

Supplementary Table S5. Comparison of the weekly frequency of meals not prepared at home among the three cohorts, in particular food consumed at restaurants and food from a local market.

	Meals at restaurant			Meals at local market		
n./week	R	SU	U	R	SU	U
0-1	100%	98%	78%	93%	100%	98%
2-3	0%	0%	4%	4%	0%	0%
4-6	0%	2%	10%	4%	0%	2%
7+	0%	0%	8%	0%	0%	0%

Supplementary Table S6. Adult BMI divided by gender. Median and IQR are reported.

Rural		Semi-Urbanized		Urban	
Males	Females	Males	Females	Males	Females
19.5 (1.7)	19.6 (2.5)	21.6 (3.4)	21.2 (6.2)	22.8 (6.4)	24.6 (4.8)
